# Smoking Dose Modifies the Association between C242T Polymorphism and Prevalence of Metabolic Syndrome in a Chinese Population

**DOI:** 10.1371/journal.pone.0031926

**Published:** 2012-03-01

**Authors:** Jiyong Ge, Zhijian Ding, Yu Song, Fangfang Wang

**Affiliations:** Department of Cardiology, Changzhou Second People's Hospital, Affiliated Nanjing Medical University, Changzhou, China; University of South Alabama, United States of America

## Abstract

**Background:**

The C242T polymorphism of the CYBA gene that encodes p22phox, a component of NADPH oxidase, has been found to modulate superoxide production. Oxidase is a major source of the superoxide anion that contributes to individual components of metabolic syndrome. We examined the relationship of the C242T polymorphism with the prevalence of metabolic syndrome in a Chinese population, taking account of consumed cigarette amounts.

**Methodology/Principal Findings:**

In 870 participants, we collected biomarkers related to metabolic syndrome and detailed history of smoking and genotyped the C242T polymorphisms. After adjustment for covariates, the CT/TT genotypes were associated with a lower risk of metabolic syndrome (P = 0.0008). The odds of having metabolic syndrome in the CT/TT participants were 0.439 (95%CI: 0.265, 0.726), while for CC participants the odds were 1.110 (95%CI: 0.904, 1.362). There was significant (P = 0.014) interaction between the C242T polymorphism and smoking status in relation to the prevalence of metabolic syndrome. For smokers who smoke no less than 25 pack-years, those with CT/TT genotypes had lower risk of metabolic syndrome as compared with CC polymorphism carriers (P = 0.015). In the multiple regression analysis, the CT/TT genotypes were significantly associated with lower serum concentration of triglycerides both in all subjects and smokers; furthermore, the CT/TT genotypes were also related to smaller waist circumference in smokers.

**Conclusions:**

Our study suggests that the C242T gene polymorphism is indeed related to the prevalence of metabolic syndrome and smoking dose might modify this association.

## Introduction

Metabolic syndrome is defined as a cluster of metabolic abnormalities that increases the risk for type 2 diabetic mellitus, coronary artery disease (CAD) and other cardiovascular diseases. The core components of metabolic syndrome are high blood pressure, central obesity, impaired fasting glucose and dyslipidemia [1]. Recently, high levels of oxidative stress and reduced production of anti-inflammatory were reported to be associated with metabolic syndrome [2].

Nicotinamide adenine dinucleotide phosphate (NADPH) oxidase, a multisubunit protein complex consisting of membrane-bound and cytosolic subunits, is a major source of superoxide anion in the vasculature [3]. The p22phox subunit is essential for activation of the NADPH oxidase system [4]. Several polymorphisms of the CYBA gene have been identified. Among these, the C242T polymorphism is located on chromosome16q24, exon4, at position 242 of the CYBA gene, and results in the amino acid substitution of tyrosine for histidine at residue 72 within one of the two heme-binding sites [5]. The functional significance of the C242T polymorphism has been related to NADPH oxidase activity with subsequent production of the superoxide anion [6], [7].

However, it is not yet clarified how it begins and how the genetic and environmental factors influence different components of metabolic syndrome. We tested the hypothesis that the C242T polymorphism in the CYBA gene is related to the prevalence of metabolic syndrome in a cross-sectional study. As smoking may influence vascular NADPH oxidase activation [8]–[11], we further examined whether smoking dose could modify the genetic effect in a Chinese population.

## Methods

### Ethics statement

The Institutional Review Board of Changzhou Second People's Hospital, Affiliated Nanjing Medical University approved the study protocol.

### Study population

All participants of the present study were recruited from the Centre for Medical Examinations in Changzhou Second People's Hospital, affiliated Nanjing Medical University of China, which provides service of comprehensive health examinations for adults who have no frank cardiovascular or non-cardiovascular diseases or acute symptoms, but may have chronic diseases such as hypertension. We did not apply any inclusion or exclusion criteria for our study. All subjects gave informed written consent.

In the periods from January 2011 to May 2011, we examined 882 subjects, who volunteered participating in our study. 12 subjects were excluded from the present analysis because of missing information on biochemical measurements (n = 7) or genotypes (n = 5). Thus, the number of subjects statistically analyzed added up to 870.

### Clinical and biochemical measurements

Experienced observers used a standardized questionnaire to collect information on smoking history (duration and daily consumption of cigarettes), consumption of alcohol, complete medical history and use of medications. Smoking habit was defined as smoking more than 1 cigarette a day for at least 1 year, or more than 360 cigarettes a year. Pack-years were calculated by multiplying the number of packs of cigarettes smoked a day by the number of years the person had smoked. The level of inhalation (pack-years) was categorised into four levels: <10 pack-years, 10∼<19 pack-years, 19∼<25 pack-years, ≥25 pack-years. Alcohol habit was defined as drinking more than twice a week, consumption of more than 50 ml of heavy liquor or 500 ml of beer on each occasion. Each participant's blood pressure was measured three times consecutively by conventional sphygmomanometry after the participants had rested for at least 5 minutes in the sitting position. These three readings were averaged for analysis. The body weight, body height, and waist and hip circumferences were measured. The body mass index was calculated as the ratio of the body weight in kilograms to the square of the height in meters, and the waist-to-hip ratio was the smallest circumference at the waist divided by the largest circumference at the hip.

After overnight fasting, a venous blood sample was drawn for the measurement of plasma glucose and serum total cholesterol, HDL cholesterol and triglycerides by an automated enzymatic method (Chemistry Analyzer AU640, Olympus Medical Engineering Company, Tokyo, Japan). LDL cholesterol concentration was calculated using the Friedewald formula [12].

### Definition of metabolic syndrome

We defined the metabolic syndrome according to the IDF criteria [13]. The metabolic syndrome was diagnosed, if a subject had abdominal obesity defined as elevated waist circumference ≥90 cm among men or ≥80 cm among women for Chinese population, and had at least two of the four following characteristics: 1) triglycerides ≥150 mg/dL (1.7 mmol/L); 2) HDL cholesterol <40 mg/dL (1.03 mmol/L) in men and <50 mg/dL (1.29 mmol/L) in women or specific treatment for these lipid abnormalities; 3) blood pressure ≥130/85 mmHg or the use of antihypertensive drugs; and 4) fasting glucose ≥100 mg/dL (5.6 mmol/L) or the presence of diabetes mellitus.

### DNA isolation and genotyping of C242T polymorphism

Whole venous blood was drawn into ethylenediamine tetraacetic acid tubes and then stored at −80°C. Genomic DNA was extracted from white blood cells using a commercially available kit and the BioRobot M48 Workstation (Qiagen Inc., Hilden, Germany) according to the manufacturer's instructions. Polymerase chain reaction (PCR) for the C242T polymorphism was performed in 384-well plates following a standard protocol [14] for TaqMan MGB probes. After the PCR was finished, endpoint fluorescence was measured, and allelic discrimination was performed using a PRISM® 7900HT Sequence Detection System (ABI) and a LightCycler® 480 Real-Time PCR System (Roche).

### Statistical analysis

For database management and statistical analysis, we used SAS software, version 9.1.3 (SAS Institute, Cary, North Carolina, USA). We reported the central tendency and spread of normally and non-normally distributed data as the mean ± standard deviation (SD) and as the median with the interquartile range, respectively. Hardy-Weinberg equilibrium was assessed using the chi-square test. We compared the means and proportions using t-test, the one-way analysis of variance (ANOVA) and the chi-square test, respectively. Logistic regression was used to examine the relationship between the C242T polymorphism and prevalence of metabolic syndrome, and to explore the interaction between smoking and the C242T polymorphism in relation to prevalence of metabolic syndrome as well. To select variables (potential confounders) for the models, we first used the univariate linear regression method to filter out variables that associate with prevalence of metabolic syndrome which included age, sex, body height, body weight, alcohol intake and heart rate. As various medications and smoking may influence the NADPH oxidase, antihypertensive medication, antidiabetic medication and smoking were also included. Therefore, the variables mentioned above were finally used as covariates in the regression models. Dominant models (major homozygous genotype as a reference) were typically used. P<0.05 was considered statistically significant.

## Results

### Characteristics of the participants

The study population included 342 (39.3%) men and 379 (43.6%) hypertensive patients, of whom 94 (24.8%) took antihypertensive medication. Men, compared with women, reported a higher proportion of smoking (P<0.0001). The characteristics of the subgroups stratified by C242T genotype among smokers and nonsmokers are shown in Table 1. LDL cholesterol concentration and male sex ratio differed significantly across genotypes among nonsmokers. While among smokers, waist circumference and serum concentration of triglycerides were greater in subjects with the CC genotype.

**Table 1:** Characteristics of the study groups stratified by genotype among smokers and nonsmokers.

Variables	Non-smokers		P	Smokers		P
	CC (n = 541)	CT+TT (n = 73)		CC (n = 217)	CT+TT (n = 39)	
Age (years)	46.4±4.4	46.4±4.2	0.90	46.6±4.4	46.5±4.3	0.85
Height (cm)	158.2±6.7	158.9±6.9	0.45	167.9±6.4	167.2±7.0	0.57
Waist circumference(cm)	82.0±9.0	82.5±8.9	0.63	87.3±9.5	84.2±8.2	0.041
Body mass index (kg/m^2^)	25.1±3.2	25.2±3.1	0.81	25.5±3.0	24.4±2.7	0.06
Waist-to-hip ratio	0.85±0.06	0.85±0.06	0.80	0.89±0.06	0.87±0.05	0.13
Heart rate (beats/min)	74.7±8.9	74.2±10.3	0.65	71.9±9.1	73.9±8.6	0.22
Systolic blood pressure (mm Hg)	131.7±16.6	129.3±14.7	0.22	134.1±14.9	137.6±17.4	0.20
Diastolic blood pressure (mm Hg)	85.2±10.0	84.7±10.0	0.67	88.7±10.2	88.1±10.1	0.12
Serum glucose (mmol/l)	5.60±1.03	5.67±1.65	0.74	5.91±1.71	6.05±1.15	0.53
Triglycerides (mmol/l)*	1.21(0.85–1.76)	1.17(0.90–1.60)	0.76	1.36(0.85–2.11)	1.19(0.76–2.04)	0.012
Total cholesterol (mmol/l)	4.79±0.90	5.00±1.02	0.07	5.08±1.04	5.01±1.08	0.71
HDL cholesterol (mmol/l)	1.31±0.28	1.29±0.29	0.73	1.23±0.32	1.31±0.39	0.16
LDL cholesterol (mmol/l)	2.86±0.66	3.03±0.72	0.04	3.09±0.75	2.91±0.75	0.18
Male sex, n (%)	78 (14.4)	19 (26.0)	0.02	210 (96.8)	35 (89.7)	0.27
Alcohol intake, n (%)	26 (4.8)	7 (9.6)	0.10	117 (53.9)	25 (64.1)	0.11
Hypertension, n (%)	209 (38.6)	24 (32.9)	0.28	123 (56.7)	23 (59.0)	0.47
Diabetes, n (%)	35 (6.5)	4 (5.5)	0.70	20 (9.2)	5 (12.8)	0.42

HDL = high-density lipoprotein; LDL = low-density lipoprotein.

* The values are the median (interquartile range).

### Genotype frequencies

The C allele frequency was 0.93 in the entire study population as well as the subgroups stratified by smoking status. The allele frequencies were in line with the Hardy–Weinberg equilibrium (χ^2^ = 2.43, P = 0.12). The allele frequencies among smokers and nonsmokers did not differ from the corresponding frequency for the entire population (P = 0.29 and P = 0.28 respectively).

### Association between C242T polymorphism and prevalence of metabolic syndrome

**Figure 1:**
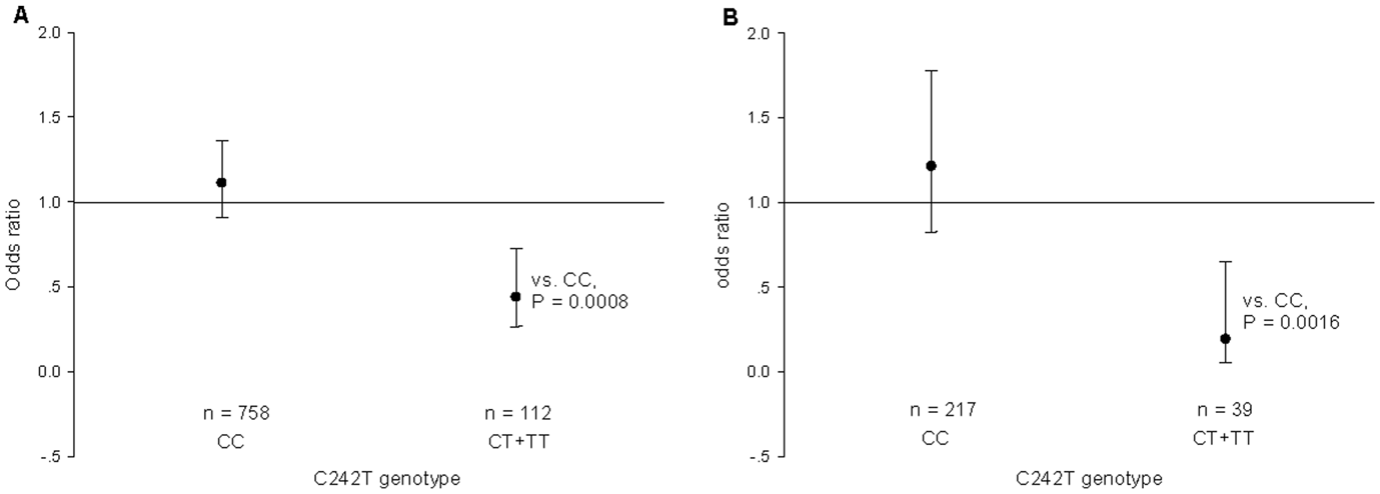
Difference of prevalence of metabolic syndrome between C242T genotypes in all participants and smokers. The odds of prevalence of metabolic syndrome in genotypic group is expressed relative to the overall risk of all participants (A) or smokers (B), and adjusted for age, sex, body height, body weight, antihypertensive medication, antidiabetic medication, current smoking, alcohol intake and heart rate. Vertical lines denote 95% confidence intervals. For each genotype, the number of participants is given.

In logistic regression, after adjustment for covariates, the CT/TT genotypes were associated with a lower risk of metabolic syndrome (P = 0.0008, Fig. 1A). The odds of having metabolic syndrome in the CT/TT participants was 0.439 (95%CI: 0.265, 0.726), while for CC participants, the odds was 1.110 (95%CI: 0.904, 1.362).

There was significant (P = 0.014) interaction between the C242T polymorphism and smoking status in relation to the prevalence of metabolic syndrome. For smokers, the subjects with CT/TT genotypes had lower risk of metabolic syndrome as compared with CC carriers (0.194 (95%CI: 0.058, 0.651) vs. 1.21 (95%CI: 0.824, 1.776), P = 0.0016, Fig. 1B). Whereas for non-smokers, no significant difference was found between subjects with CT/TT genotypes and CC carriers (0.571 (95%CI: 0.324, 1.005) vs. 1.07 (95%CI: 0.841, 1.365), P = 0.06). No such interaction was found between C242Tpolymorphism and gender (P = 0.068). In further stratified analysis, we observed a significant association of C242T polymorphism with the prevalence of metabolic syndrome in male smokers (P = 0.016), while such relationship was not observed in male non-smokers (P = 0.09).

**Figure 2:**
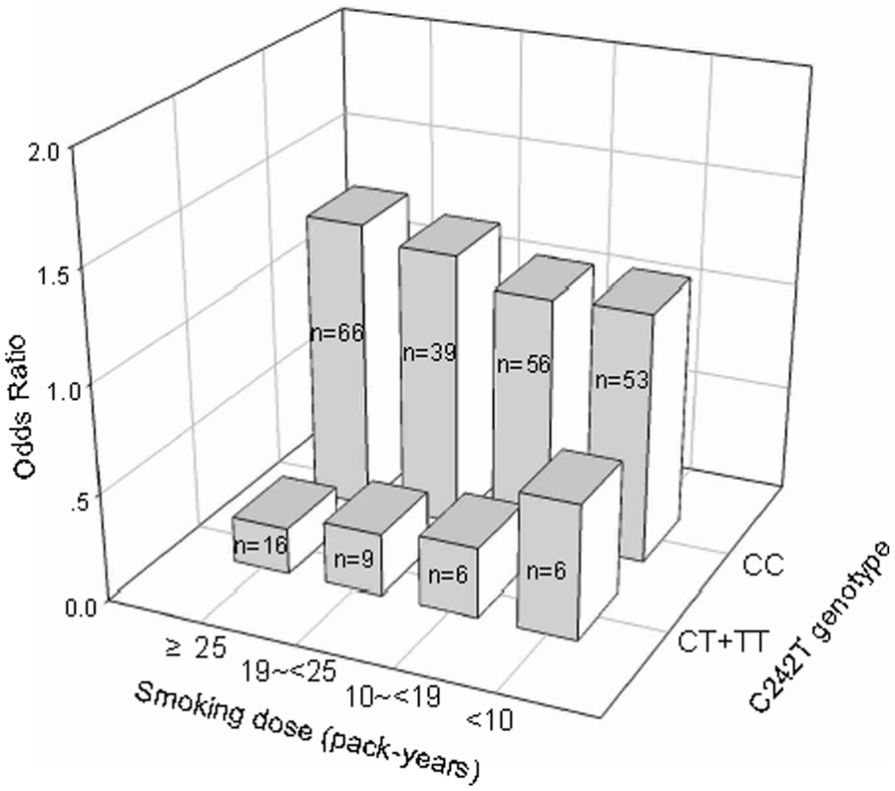
Difference of prevalence of metabolic syndrome between C242T genotypes in different smoking dose groups. The odds of prevalence of metabolic syndrome in genotypic group is expressed relative to the participants in each smoking dose group, and adjusted for the same covariates as in the Figure 1.

The significant association between the C242T polymorphism and the prevalence of metabolic syndrome was further found in subjects who smoke no less than 25 pack-years (0.217 (95%CI: 0.026, 0.474) vs. 1.345 (0.614, 2.946), P = 0.015, Fig. 2). While no such relationship was found in <10 pack-years group (0.623 (95%CI: 0.070, 5.574) vs. 1.167 (95%CI: 0.517, 2.630), P = 0.84, Fig. 2), 10∼<19 pack-years group (0.322 (95%CI: 0.037, 2.789) vs. 1.155 (95%CI: 0.538, 2.480), P = 0.51, Fig. 2) or 19∼<25 pack-years group (0.288 (95%CI: 0.058, 1.429) vs. 1.274 (95%CI: 0.597, 2.719), P = 0.78, Fig. 2).

### Association between C242T polymorphism and the individual components of the metabolic syndrome

In the multiple regression analysis, the CT/TT genotypes were significantly associated with lower serum concentration of triglycerides both in all subjects and smokers, furthermore, the CT/TT genotypes were also related to smaller waist circumference in smokers. While for non-smokers, there was no such relationship (Table 2).

**Table 2:** Multiple regression analysis of C242T genotypes.

Variables	Genotypes	All subjects		Smokers		Non-smokers	
		Mean ± SE*	P	Mean ± SE#	P	Mean ± SE#	P
Waist circumference (cm)	CC	83.6±0.32	0.37	87.4±0.60	0.006	82.0±0.37	0.88
	CT+TT	82.7±0.85		83.4±1.57		82.2±1.01	
Systolic blood pressure (mm Hg)	CC	132.3±0.55	0.95	134.1±0.99	0.27	131.6±0.66	0.39
	CT+TT	132.6±1.47		137.4±2.59		130.0±1.77	
Diastolic blood pressure (mm Hg)	CC	86.2±0.34	0.58	88.8±0.65	0.38	85.2±0.40	0.79
	CT+TT	86.8±0.91		91.1±1.71		84.9±1.07	
Serum HDL cholesterol concentration (mmol/L)	CC	1.28±0.01	0.21	1.23±0.03	0.17	1.31±0.01	0.99
	CT+TT	1.32±0.03		1.31±0.06		1.31±0.03	
LogTG concentration (mmol/L)	CC	0.32±0.02	0.04	0.49±0.04	0.02	0.25±0.02	0.65
	CT+TT	0.20±0.05		0.11±0.12		0.22±0.06	
Serum glucose concentration (mmol/L)	CC	5.70±0.04	0.58	5.92±0.09	0.46	5.60±0.04	0.59
	CT+TT	5.77±0.11		6.05±0.25		5.67±0.12	

HDL = high density lipoprotein.

LogTG = Log transformation of the triglycerides.

* Adjusted for sex, age, body height, body weight, antihypertensive medication, antidiabetic medication, current smoking, alcohol intake and heart rate.

# Adjusted for sex, age, body height, body weight, antihypertensive medication, antidiabetic medication, alcohol intake and heart rate.

After exclusion of 111 patients on various medications that may modulate NADPH oxidase activities, our findings remained unaltered (data not shown).

## Discussion

To our knowledge, this is the first study to investigate the relationship between the C242T polymorphism and the prevalence of metabolic syndrome in a Chinese population. Our main findings were that the CT/TT genotypes of the C242T polymorphism were associated with a lower risk of metabolic syndrome and that smoking habit might modify this association. The significant genotype-phenotype association was especially marked in smokers who smoked no less than 25 pack-years.

In recent years, the C242T polymorphism of the CYBA gene has been widely discussed in the context of cardiovascular diseases, however, the results were conflicting [7], [15]–[18]. The C242T polymorphism of the CYBA gene that codes p22phox had been found to modulate superoxide production. Functionally, superoxide production has been linearly associated with the C242T genotypes, being highest in CC, and lower in CT/TT genotypes [7]. Meanwhile, a latest meta-analysis conducted by Fang et al [19] demonstrated that the T allele of the C242T polymorphism was related to reduced CAD risk only in Asian population, which indicated that differences in the ethnicity may cause disparities in results. Accordingly, we also found a significant protective effect of the T allele for metabolic syndrome in a Chinese population, and such effect was mainly focused on serum concentration of triglycerides. Several studies [20], [21] had demonstrated that triglyceride correlated strongly with leukocyte production of reactive oxygen species (ROS). This might be due to oxidative stress by the modification of lipoprotein. It is known that perioxidized fatty acids can activate phospholipase A_2_ [22], which may increase the release of arachidonic acid from leukocyte membrane and therefore contribute to the activation of NADPH oxidase.

Cigarette smoke contains many xenobiotics, including free radicals and oxidants that can increase lipid peroxidation. One previous study [23] indicated that cigarette smoke contains about 10^14^ free radicals per inhalation. Specific biomarkers of lipid peroxidation were reported to be associated with the number of cigarettes smoked daily, with lipid peroxidation increasing with the number of cigarettes smoked [24]. Increased superoxide production that derived from smoking leads to the inactivation of nitric oxide, thus contributing to endothelial dysfunction and predisposing to the development of metabolic syndrome [25], [26]. Since membrane-associated NADPH oxidase is the primary physiological producer of reactive oxygen species, including superoxide [27], p22phox is the primary enzyme interface between environmental oxidants and host organism, the genetic susceptibility ascribable to this enzyme family is expected to be associated with different dose levels of exposed xebobiotics. In the present study, we studied the difference in susceptibility to metabolic syndrome in terms of C242T polymorphism of the CYBA gene, taking account of consumed cigarette amounts. This indicates that the genetic susceptibility ascribe to CYBA 242 C plays an important role in occurrence of metabolic syndrome, especially in participants who smoke greater than 25 pack-year. Similar results have been reported by Fan et al [28], who found that the C242T polymorphism of the CYBA gene was related to the brachial artery FMD response in young healthy Finnish adults, and that body adiposity and smoking status may modify this association. Contrary to these findings, Niemiec et al [29] reported the 242T allele interacted with cigarette smoking and hypercholesterolemia to increase the risk of CAD and this risk is probably associated with the cumulative/synergistic effect of the 242T allele and both the traditional risk factors, however, some recent studies [30], [31] have failed to find any effect of the C242T polymorphism either on venous endothelial function in healthy individuals or on forearm endothelium-dependent vasodilatation in subjects with hypercholesterolemia. Differences in vascular territories and sample sizes may explain such discrepancies.

The components of the antioxidant defense system maintain the levels of ROS in the normal cellular homeostatic range. That is, under normal conditions this system reduces excess ROS to minimize damage, yet still allows for ROS required in intracellular redox signaling. However, certain pathological conditions, such as NADPH oxidase activation, hyperglycemia, and hyperlipidemia, have been shown to promote oxidative stress through elevated ROS production and/or reduced antioxidant defense [32]. Several lines of evidence have suggested that fat accumulation is correlated with high oxidative stress state in human populations [33]. In consistent with these evidence,the present study revealed that the CC genotype was related to larger waist circumference in smokers, that may resulted from the excessive activation of NADPH oxidase in smokers with elevated levels of oxidative stress [34]. But the direct cause of this increase in waist circumference with elevated oxidative stress is still not entirely clear.

The interaction by smoking might be potentially confounded by gender-specificity for males were with more proportion of smoking habit. However, the interaction terms between explanatory variables and sex were non-significant, and similar results were found in males compared to the whole subjects. Meanwhile, there were also no statistical differences between genetic variations and prevalence of metabolic syndrome after stratified by sex (data not shown).

Our study had to be interpreted within the context of its limitations. First, our study sample was not randomly selected or general population, selection bias was possible. Although the majority of smokers in our participants are male, it was in line with the Chinese national condition [35] and previous WHO reports (61% of men are reported to be current smokers in China compared with only 4.2% of women) [36], and did not have major influence over the main findings in present study. Second, we did not directly measure the biochemical indices related to superoxide production to verify our hypothesis. Third, the frequency of T allele in Asian population is relatively small than non-Asian population, so the relatively small sample size in the lower smoking dose groups in T allele carriers may have limited the statistical power. Fourth, we did not collect information on time of exposure to tobacco snuff. Finally, since our study was cross-sectional, no causal inference could be taken.

In conclusion, our study suggests that the prevalence of metabolic syndrome is indeed related to the C242T gene polymorphism in relation to the amount of cigarette inhalation. However, the predicative value of smoking and C242T polymorphism for metabolic syndrome in the Chinese population remains to be elucidated.

## References

[1] Evangelopoulos AA, Vallianou NG, Panagiotakos DB, Georgiou A, Zacharias GA (2008). An inverse relationship between cumulating components of the metabolic syndrome and serum magnesium levels.. Nutr Res.

[2] Rao VS, Nagaraj RK, Hebbagodi S, Kadarinarasimhiah NB, Kakkar VV (2010). Association of inflammatory and oxidative stress markers with metabolic syndrome in asian indians in India.. Cardiol Res Pract.

[3] Ye S, Wang Y, Jiao F, Zhang H, Lin C (2011). The role of NADPH oxidase in multi-walled carbon nanotubes-induced oxidative stress and cytotoxicity in human macrophages.. J Nanosci Nanotechnol.

[4] Ushio-Fukai M (2006). Localizing NADPH oxidase-derived ROS.. Sci STKE.

[5] Inoue N, Kawashima S, Kanazawa K, Yamada S, Akita H (1998). Polymorphism of the NADH/NADPH oxidase p22phox gene in patients with coronary artery disease.. Circulation.

[6] Guzik TJ, West NE, Black E, McDonald D, Ratnatunga C (2000). Functional effect of the C242T polymorphism in the NAD(P)H oxidase p22phox gene on vascular superoxide production in atherosclerosis.. Circulation.

[7] Wyche KE, Wang SS, Griendling KK, Dikalov SI, Austin H (2004). C242T CYBA polymorphism of the NADPH oxidase is associated with reduced respiratory burst in human neutrophils.. Hypertension.

[8] Jaimes EA, DeMaster EG, Tian RX, Raij L (2004). Stable compounds of cigarette smoke induce endothelial superoxide anion production via NADPH oxidase activation.. Arterioscler Thromb Vasc Biol.

[9] Raij L, DeMaster EG, Jaimes EA (2001). Cigarette smoke-induced endothelium dysfunction: role of superoxide anion.. J Hypertens.

[10] Furukawa S, Fujita T, Shimabukuro M, Iwaki M, Yamada Y (2004). Increased oxidative stress in obesity and its impact on metabolic syndrome.. J Clin Invest.

[11] Sonta T, Inoguchi T, Tsubouchi H, Sekiguchi N, Kobayashi K (2004). Evidence for contribution of vascular NAD(P)H oxidase to increased oxidative stress in animal models of diabetes and obesity.. Free Radic Biol Med.

[12] Friedewald WT, Levy RI, Fredrickson DS (1972). Estimation of the concentration of low-density lipoprotein cholesterol in plasma, without use of the preparative ultracentrifuge.. Am Assoc Clin Chem.

[13] Alberti KG, Zimmet P, Shaw J (2006). Metabolic syndrome-a new world-wide definition. A Consensus Statement from the International Diabetes Federation.. Diabet Med.

[14] Livak KJ (1999). Allelic discrimination using fluorogenic probes and the 5′ nuclease assay.. Genet Anal.

[15] He MA, Cheng LX, Jiang CZ, Zeng HS, Wang J (2007). Associations of polymorphism of P22(phox) C242T, plasma levels of vitamin E, and smoking with coronary heart disease in China.. Am Heart J.

[16] Zafari AM, Davidoff MN, Austin H, Valppu L, Cotsonis G (2002). The A640G and C242T p22(phox) polymorphisms in patients with coronary artery disease.. Antioxid Redox Signal.

[17] Arca M, Conti B, Montali A, Pignatelli P, Campagna F (2008). C242T polymorphism of NADPH oxidase p22phox and recurrence of cardiovascular events in coronary artery disease.. Arterioscler Thromb Vasc Biol.

[18] Macias-Reyes A, Rodriguez-Esparragon F, Caballero-Hidalgo A, Hernandez-Trujillo Y, Medina A (2008). Insight into the role of CYBA A640G and C242T gene variants and coronary heart disease risk. A case-control study.. Free Radic Res.

[19] Fang S, Wang L, Jia C (2010). Association of p22phox gene C242T polymorphisms with coronary artery disease: A meta-analysis.. Thromb Res.

[20] Bae JH, Bassenge E, Kim KB, Kim YN, Kim KS (2001). Schwemmer M. Postprandial hypertriglyceridemia impairs endothelial function by enhanced oxidant stress.. Atherosclerosis.

[21] Araujo FB, Barbosa DS, Hsin CY, Maranhão RC, Abdalla DS (1995). Evaluation of oxidative stress in patients with hyperlipidemia.. Atherosclerosis.

[22] Sevanian A, Wratten ML, McLeod LL, Kim E (1988). Lipid peroxidation and phospholipase A2 activity in liposomes composed of unsaturated phospholipids: a structural basis for enzyme activation.. Biochim Biophys Acta.

[23] Church DF, Pryor WA (1985). Free-radical chemistry of cigarette smoke and its toxicological implications.. Environ Health Perspect.

[24] Brude IR, Drevon CA, Hjermann I, Seljeflot I, Lund-Katz S (1997). Peroxidation of LDL from combined-hyperlipidemic male smokers supplied with omega-3 fatty acids and antioxidants.. Arterioscler Thromb Vasc Biol.

[25] Cozma A, Orăşan O, Sâmpelean D, Fodor A, Vlad C (2009). Endothelial dysfunction in metabolic syndrome.. Rom J Intern Med.

[26] Simão AN, Lozovoy MA, Simão TN, Venturini D, Barbosa DS (2011). Immunological and biochemical parameters of patients with metabolic syndrome and the participation of oxidative and nitroactive stress.. Braz J Med Biol Res.

[27] Dutta S, Rittinger K (2010). Regulation of NOXO1 activity through reversible interactions with p22phox and NOXA1.. PLoS One.

[28] Fan M, Raitakari OT, Kähönen M, Juonala M, Hutri-Kähönen N (2007). CYBA C242T gene polymorphism and flow-mediated vasodilation in a population of young adults: the Cardiovascular Risk in Young Finns Study.. J Hypertens.

[29] Niemiec P, Zak I, Wita K (2007). The 242T variant of the CYBA gene polymorphism increases the risk of coronary artery disease associated with cigarette smoking and hypercholesterolemia.. Coron Artery Dis.

[30] Fricker R, Hesse C, Weiss J, Tayrouz Y, Hoffmann MM (2004). Endothelial venodilator response in carriers of genetic polymorphisms involved in NO synthesis and degradation.. Br J Clin Pharmacol.

[31] Schneider MP, Hilgers KF, Huang Y, Delles C, John S (2003). The C242T p22phox polymorphism and endotheliumdependent vasodilation in subjects with hypercholesterolaemia.. Clin Sci (Lond).

[32] Madamanchi NR, Vendrov A, Runge MS (2005). Oxidative stress and vascular disease.. Arterioscler Thromb Vasc Biol.

[33] Couillard C, Ruel G, Archer WR, Pomerleau S, Bergeron J (2005). Circulating levels of oxidative stress markers and endothelial adhesion molecules in men with abdominal obesity.. J Clin Endocrinol Metab.

[34] Keaney JF, Larson MG, Vasan RS, Wilson PW, Lipinska I (2003). Obesity and systemic oxidative stress: clinical correlates of oxidative stress in the Framingham Study.. Arterioscler Thromb Vasc Biol.

[35] Lv J, Liu Q, Ren Y, Gong T, Wang S (2011). Socio-demographic association of multiple modifiable lifestyle risk factors and their clustering in a representative urban population of adults: a cross-sectional study in Hangzhou, China.. Int J Behav Nutr Phys Act.

[36] Hitchman SC, Fong GT (2011). Gender empowerment and female-to-male smoking prevalence ratios.. Bull World Health Organ.

